# Membrane Packing Problems: A short Review on computational Membrane Modeling Methods and Tools

**DOI:** 10.5936/csbj.201302014

**Published:** 2013-04-07

**Authors:** Björn Sommer

**Affiliations:** a Bio-/Medical Informatics Department, Bielefeld University, Universitätsstraße 25, 33615 Bielefeld, Germany

## Abstract

The use of model membranes is currently part of the daily workflow for many biochemical and biophysical disciplines. These membranes are used to analyze the behavior of small substances, to simulate transport processes, to study the structure of macromolecules or for illustrative purposes. But, how can these membrane structures be generated?

This mini review discusses a number of ways to obtain these structures. First, the problem will be formulated as the Membrane Packing Problem. It will be shown that the theoretical problem of placing proteins and lipids onto a membrane area differ significantly. Thus, two sub-problems will be defined and discussed. Then, different – partly historical – membrane modeling methods will be introduced. And finally, membrane modeling tools will be evaluated which are able to semi-automatically generate these model membranes and thus, drastically accelerate and simplify the membrane generation process. The mini review concludes with advice about which tool is appropriate for which application case.

## Introduction

The basis for many biophysical and biochemical *in silico* experiments is a model membrane. Experiments may be used to assemble protein-membrane systems to verify and simulate the structure of proteins or to theoretically evaluate values derived by wet lab experiments (e.g., the area per lipid, the membrane thickness, the viscosity with respect to temperature changes and the transition between different phases). The behavior of small organic and anorganic substances can be evaluated as well as the permeability, active and passive transport processes. And of course these model membranes can also be used for illustrative purposes.

These structures have to be generated using fragmentary knowledge about the composition of biological membranes since it is not possible to acquire the complete membrane structure with presently used microscopy or spectroscopy methods. As there is no standard procedure, there are many different ways to acquire these initial structures. Basically, three different methods can be distinguished to generate a model membrane.

1. Structure Recycling: First, they may be “recycled”. This means, they can be taken from one's own previous in silico experiments or downloaded from websites and other repositories. They can also be taken from the supplement of published material. The advantage is that the membrane layer is already equilibrated. However, it is not always possible to find a simulated membrane showing the composition needed for a computational experiment.
2. Manual Structure Generation: These structures can be generated by scripting or by composing them using a text editor. For example, there are some software tools available which, although they were not originally developed to generate membranes, may be used in conjunction with a script to generate membranes. Related approaches will be summarized under the chapter Membrane Modeling Methods.
3. (Semi-)Automated Structure Generation: In the past few years new software tools were developed which were optimized for the modeling of membranes..These tools will be introduced towards the end of this mini-review.

This mini review explicitly cannot provide an extensive summary of all tools and methods available today. But to the knowledge of the author, this publication covers the most important ones. This discussion will primarily focus on the creation of rectangular membrane patches. This work concludes with suggestions for the appropriate tool for a given application case.

But first, the theoretical background of membrane modeling has to be discussed. This background was previously introduced in our publication from 2011 [1].

### From Nature to Model

Nature is our role-model. Since the Fluid Mosaic Model was introduced in the 1970s, many different observations have approved and extended this theoretical membrane model [2]. It is known that the membrane is not a rigid structure. It is a highly dynamic culmination of different specific biological entities such as proteins and lipids. These entities are in continuous fast-motion. Lipids change their positions and structure. Some of them are changing from the external side of a membrane bilayer to the internal side [3]. Proteins bind with lipids or other proteins located inside the membrane. Substances are transported from one side of the membrane to the other. And of course, while the elements of the membrane are changing, the shape of the membrane changes too. Lipid rafts float like small islands through the membrane sea [4] and membrane caves are formed, ready to enclose cargo, separate from the membrane and transport the cargo through the cytosol.

Very complex problems arise when the membrane modeler takes all these different aspects into account. How is it possible to simulate all the biological, chemical and physical aspects of all those membrane components? The physicist might wish to use quantum mechanical approaches, but then it would be nearly impossible to simulate the structure of a single protein. Therefore, approaches exist combining molecular dynamic and quantum mechanical approaches [5]. Although pure quantum mechanical approaches are applied to a problem, it is still not possible to prove that the resulting *in silico* structures absolutely represent their real state *in vivo*.

A number of different approaches exist to simulate membrane structures, trying to reproduce experimentally derived values in the wet lab. The initial guess could be that these simulation approaches could be used to generate an initial membrane model. This approach will be shortly discussed as the *self-assembly method*. The problem is that the intended structure will often not be achieved. If, for example, the membrane modeler wants to generate a rectangular membrane patch, a system could be created containing water and a number of lipids randomly placed in a cubic box. If a Molecular Dynamics (MD) simulation is now applied to this system, the lipids will usually tend to form micelles instead of generating rectangular structures. Here is a major dilemma because the rectangular membrane patch is a fragment which usually does not occur in nature. But it is applied to many different application cases in order to examine a small fragment of reality as the molecular simulation of a complete cell membrane lies beyond the scope of present computer technology.

An alternate approach is to generate a stiff starting structure using different techniques. Usually, in the next step, it will need to be proven that the resulting *in silico* structure is stable over time by applying a molecular simulation. In this case, the experimentalist will need force fields as well as the coordinate information of the lipids. The force fields describe the physicochemical behavior of lipids, including torsion angles and atomic forces. This information is only available for a small amount of lipids. However, all actual simulation environments such as CHARMM, GROMACS, or NAMD, provide force fields compatible with lipid simulations [6–8].

### Packing Problems

If the process of creating a membrane is broken down into its most basic task(s), it can be understood as a packing problem. These are known from a wide range of economical and ecological applications. One example is the logistic sector, where the task is to optimize the relation between space and cargo [9]. Packing problems are known to be NP-hard [10], and solving them is a complex task.

Following the definition of our previous publication, the issue is described as the *Membrane Packing Problem (MPP)* [1]. Two sub-problems have to be distinguished. First, the *Lipid Packing Problem (LPP)* and second the *Protein Packing Problem (PPP)*.

- *(LPP): Lipid Packing Problem:* How can one or multiple lipid models be optimally packed onto a monolayer or bilayer?
- *(PPP): Protein Packing Problem:* How can one or multiple protein models be optimally packed onto a monolayer or bilayer?
- *(MPP): Membrane Packing Problem:* How can one or multiple lipid and protein models be optimally packed together onto a monolayer or bilayer with respect to LPP and PPP?

In the following chapters it will be shown that it is important to make the distinction as the packing of lipids onto a membrane area differs significantly from the insertion or attachment of a membrane protein.

## Membrane Modeling Methods

A number of traditional membrane modeling methods should be discussed: the Grid-based, the Replacement, the Insertion and the Self-assembly method. As previously mentioned, these – partly very time-consuming – methods were especially relevant in the past. Then there were no software tools freely available which were optimized for the membrane generation process. But there are a number of relevant PPP methods listed in the following sections which are not integrated into semi-automatic Membrane Modeling Tools discussed towards the end of this manuscript.

Often, molecular modeling packages had to be used which provided a scripting interface. The user had to write a script which created an initial membrane model, as discussed in the next section. An alternative way was to write small customized tools. Often they were never published by the authors and are therefore not freely available. All methods discussed here are partly used to create pure membranes as well as protein/membrane complexes.

### Grid-based Methods

The idea of the Grid-based method is quite simple: the molecules are placed along a virtual grid, representing the horizontal layer located between the two layers of a bilayer membrane. If 64 lipid models should be placed at one layer, a virtual grid with a resolution of 8x8 is generated, and a molecule is placed at each intersecting point of this grid.

**Figure 1:**
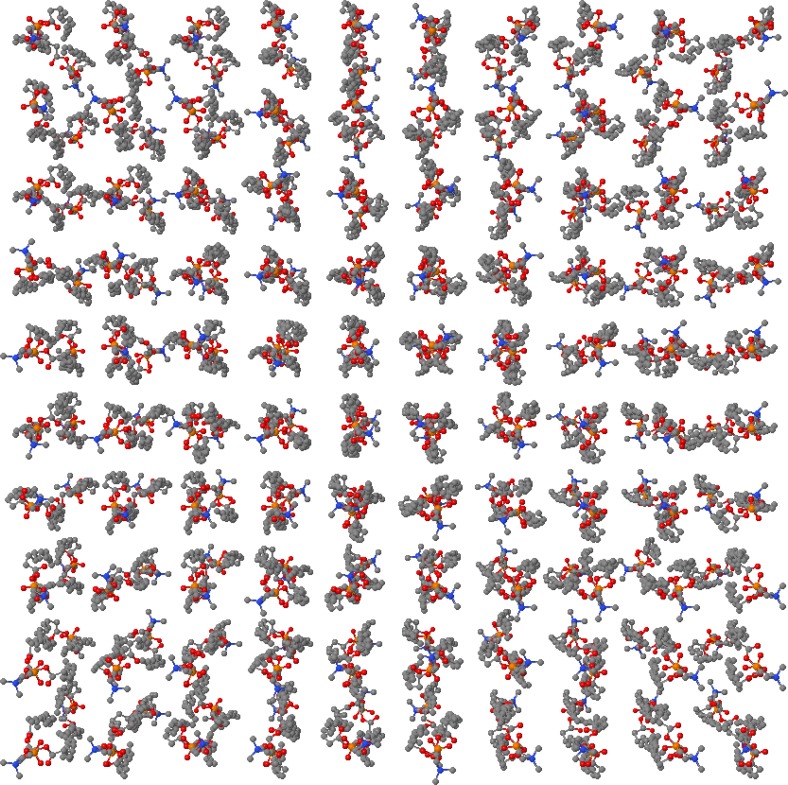
The result of the MOE Membrane Grid script as shown in Jmol.

A typical script should be shortly explained. It was re-implemented by the author of this mini review based on the method published by Krüger and Fischer [16]. For this purpose, MOE (Molecular Operating Environment) was used. A number of alternative tools could also be applied. MOE is a commercial and well-established modeling tool which is used, for example, in drug discovery [17]. MOE is not originally intended to create membrane structures. But it is possible to do so by writing a script, using the scripting vector language (SVL). The applied script implements two loops. The first loop operates along the Z-axis, the second loop along the X-axis. A defined number of lipids are placed along each axis with respect to the user-defined distance. The user may also choose if a monolayer or bilayer should be created. The resulting DPPC lipid with ten lipids per row and a distance of 10 Å can be seen in Figure 1.

By examining the result in Figure 1 it is obvious that it is not able to meet biological structural expectations, because the grid is too regular. The resulting structure is very vague and ignores geometrical or chemophysical aspects. All molecules in the resulting structure feature the same distance from the neighboring lipids. Therefore, in the best case, a number of minimization steps will be needed to generate a membrane model compatible with molecular simulations. But in the worst scenario, the gaps between the lipids might be too large. This state will usually result in a membrane, where the water molecules, added during the solvation process, fill the bilayers′ gaps. During the simulation, the water-filled gaps will expand and in this way they will usually destroy the bilayer structure. Of course, the structure of the membrane in Figure 1 could be improved by choosing initial grid values meeting experimental expectations concerning the area per lipid. It will be densely packed but it can be surmised that the atoms of the lipids will collide or even intersect. An add-on minimization might rectify the situation, although some simulation packages may have extreme difficulty with these intersecting structures. However, to prevent as many collisions as possible, it is a good idea to use lipid models with stretched-out tails. It can be summarized that this is a quick method to generate an initial structure for those researchers familiar with a molecular modeling environment providing scripting interfaces. But in the next step it might be troublesome because the energy minimization might take too long or could even fail. In both cases it would be a better approach to use a packing which tries to avoid collisions.

In addition, this membrane is only homogeneous. The MOE Membrane Grid script could be extended or the replacement method discussed in the following section could be applied if different types of lipids have to be inserted.

The membrane modeling tools discussed in the next chapter could be practical for the researcher who has not previously written a script or who is unfamiliar with a similar tool.

An interesting method combining a Grid-based method with protein placement was discussed by Kandt, Ash and Tieleman. They applied a widely-spaced grid of lipids, similar to the one shown in Figure 1, while placing a protein in the grid′s center. Then, they shrank the grid around the protein, moving the lipids closer to the edge of the protein, until the intended area per lipid was reached [12].

### Replacement Methods

Initially, Pastor et al. described the *Replacement method* [18]. Different publications on this process are summed up in the following section:

1. If the final membrane structure should contain a single protein or peptide, this molecule may be used as the starting structure [19, 20]. During the following steps (Step 2 to 7), this molecule defines the center of the membrane and it remains fixed at its initial position.
2. The starting point of the lipid membrane is a single lipid model which is used during the generation process. But instead of using the regular atomic structure of this lipid, it is represented by a sphere. This object may be the representative for a headgroup [18], a Lennard-Jones sphere of 4.8 Å radius corresponding to its average cross-sectional area [19], a van der Waals radius sphere [20] or spherical beads with an approximated size of the polar headgroups [21].
3. Now, the spheres representing the lipids are distributed onto a plane (by omitting protein in the center from step 0, if present). The placement of the lipids may follow a regular hexagonal [18] or octagonal arrangement [19], or they are just randomly distributed onto the plane [20, 21].
4. This step might not be necessary if homogeneous bilayers are constructed [18]. But the packing of spheres representing the lipids might have to be optimized if heterogeneous bilayers are constructed. For this purpose, different approaches may be used. This might be an MD simulation [19, 20] as well as a shrinking process using the steepest descent minimization algorithm [21, 22]. During the different repositioning processes, the vertical positions of the sphere usually remain fixed. But it may also be that the vertical movement is possible within a short range [20].
5. Until now the spheres were utilized to substitute the atomic structure of the lipids. Now that the distribution process is finished, these spheres have to be substituted by the original lipid structures. These are randomly chosen from a trajectory, which is a library containing a limited number of different conformations of a lipid chain [18]. These trajectories have been previously created by simulations, often taken from already published and verified data [23, 24]. If the orientation of the lipids is not uniform, the lipids may also be rotated randomly in a range of 0 to 360° in the X/Z-plane and tilted in a range from 0 to 45° with respect to the bilayer which is normal during the substitution process [18]. In this context, the placing process does not usually take collision detection into account. For this reason, there will be steric collisions inside the generated membrane model if it is imported into a molecular simulation environment. This may be problematic if a molecular simulation should be invoked afterwards.
6. For this purpose, different methods are now applied which are all similar or equal to minimization processes. During the energy minimization of the membrane structure, the radii of the atoms grow from 1 to 100% [18]. Another approach is that the lipid structures (and optionally also the protein or peptide in the center) remain stiff while they are systematically horizontally moved and tilted [19]. Sometimes this method can also be combined with an increasing process of the atom radii [20]. Sometimes the Steepest Descent (SD) minimization algorithm can also be used for this task [21].
7. These optimization methods reduce the bad contacts but usually they do not disappear (for example, a decrease of factor 2 is observed [19]). To solve the dilemma of the remaining collisions, a number of atomistic energy minimizations has to be applied.
8. Now, the model membrane is ready for the equilibration process. Therefore, all constraints, like the fixation to a position in the X/Z plane, are removed and the molecules are able to move and interact freely.
9. Finally, the initial structure is complete and the molecular simulation, e.g., an MD simulation, can be 1. started.

### Insertion Methods

The Insertion method is often used to integrate larger molecules such as proteins or peptides into a previously generated bilayer (PPP). Therefore, the subsequent – partly historical – examples roughly discuss protein insertion methods with focus on the surrounding bilayer. In contrast with the previous method (Replacement method, Step 0), first the membrane is generated, and then the protein is inserted into the surrounding layer. A short overview of methods will follow.

1. A combination of the insertion method with the replacement method was discussed by Shen, Bassolino and Stouch. The base was a previously-published starting structure of a well-equilibrated bilayer membrane. At each layer, two lipid models were removed from the center. But the hole inside the bilayer was not large enough to accommodate the peptide. To increase the radius, weak cylindrical repulsive forces were appended to the hole during the subsequent minimization and MD simulation process. As soon as the radius of the whole was large enough, the peptide was inserted. But because the atoms of the lipid and those of the peptide were still colliding at some positions, the system was minimized again. Finally, after all collisions were eliminated, the equilibration and MD simulation could follow [25].
2. A classical approach was discussed by Kandt, Ash and Tieleman. The protein is inserted into a pre-equilibrated bilayer and overlapping lipids are removed based on cut-off lipid-protein distances. The aforementioned publication lists a number of approaches with this strategy. As the authors state, the main disadvantage of this approach is that the distances between the remaining lipids and the protein are irregular. Therefore, difficulties might arise when starting the simulation because the initial simulation box size might already shrink during the first simulation steps [12].
3. A slightly different approach was applied by Tieleman and Berendsen. Initially, a bilayer containing 64 lipids on both sides was created using a grid-like positioning and random rotating method. After the short solvation and simulation process (using periodic boundary conditions) the bilayer was multiplied by 4, resulting in a membrane containing 256 lipids at each side. Then, two methods were used to insert a protein into the membrane.
  - First, the protein was inserted into the membrane without removing intersecting lipids after which an abstract two-dimensional grid was laid on top of the membrane area. Each quadratic area was checked for the coexistence of the protein with lipids. All lipids affecting the quadratic area were removed. The advantage of this approach is that the resulting membrane did not exhibit any atomic collisions. On the downside, it showed large distances between the lipids and protein.
  - Alternatively, Tieleman and Berendsen used the previously-discussed approach but with the exception of allowing minor intersections. After the initial structure was created, a number of energy minimization attempts combined with force-related investigations and the removal of some intersecting lipids were needed. It was more complicated to achieve then the first approach but the resulting structure did not show large gaps between lipids and proteins [26].
4. Faraldo-Gómez, Smith and Sansom began with the generation of a solvated and equilibrated lipid bilayer. Next, the solvent-accessible surface area of the protein was computed. The resulting shape was used in the following process as the protein′s placeholder. Then, all lipids were removed if their headgroup P atom was intersecting a cylinder-shaped region where the protein should be placed. In the next step the protein was inserted into the bilayer and only some lipid tails extended into its region. The small number of intersections were eliminated during the following MD simulations [27].
5. Instead of placing a protein, it is also possible to place other specific molecules into a virtual membrane area. One example was discussed by Deleu et al. In this case, a surfactin was fixed in the membrane′s center. Then, a new lipid entered the membrane area and moved along the X-axis in small increments less than an Ångstrom. At each step, the lipid slightly rotated, changed its vertical position and tilted. Meanwhile, the energy of each composition was calculated and the one with the lowest energy was kept using a tool called HYPERMATRIX. Then, the next lipid was added and went through the aforementioned procedure [28, 29].
6. Yesylevskyy introduced a plugin called *ProtSqueeze*. It was initially developed to be used in conjunction with VMD and the source code could be used with virtually any other program supporting plugins or scripts. The workflow of ProtSqueeze will be shortly summarized [30].
  - A pre-equilibrated bilayer has to be used and a protein has to be pre-aligned into the bilayer. For this purpose ProtSqueeze is able to use OPM structures for the pre-alignment [15]. Alternatively, ProtSqueeze provides a number of scripts, which try to place the protein in relation to the bilayer based on its residues defining the two membrane-water interfaces. Afterwards, all lipids are deleted which intersect the protein based on the steric clashes. The user may define a percentage value which varies the grade of intersection.
  - Now, the squeezing process starts. At this stage, the structure of the protein shrinks until all steric clashes are eliminated. A special method is used to take any possible tilts of the protein into account.
  - Finally, the shrunken structure of the protein has to be expanded again. At each sub-step, the protein is slightly stretched, followed by an energy minimization of the surrounding lipids to remove possible new overlaps. Step 3 is repeated until the protein′s original size is reached, of course avoiding atomic collisions.
7. A very similar approach is today part of the GROMACS simulation suite. It is called g_membed [13].
8. Jo, Kim and Im developed an insertion method in conjunction with a web interface (to be introduced later in the Membrane Modeling Tool section) [31]. A hole is created inside a pre-equilibrated bilayer by applying repulsive radial forces around the center of the protein. For this purpose, lipid bilayers were generated, equilibrated, and collected in a library which contains membranes of various sizes and hole radii. Based on the size of the protein, an appropriate bilayer is chosen and the position of the protein-surrounding lipids is adjusted based on the repulsive radial forces.
9. Staritzbichler et al. introduced a methodology called GRIFFIN, which improves the protein insertion process. Lipids and water molecules of a pre-equilibrated system are removed corresponding to the volume of the protein. The protein is placed in the space created. However, still non-protein atoms intersect the protein. Then, a grid-based implicit force field is used during a subsequent Molecular Dynamics simulation. Atoms of non-protein molecules lying inside the protein′s volume are confronted with an outward force which moves them outside the protein′s volume, whereas the external non-protein atoms are stirred by van der Waals and electrostatic forces. The big advantage is that the overall volume and density of the simulation box is preserved and the surrounding non-protein molecules are smoothed to the surface of the protein [14].
10. Another option placing a protein inside a membrane is to acquire protein pre-alignment information from specific databases. PDB_TM and OPM provide information which enables the alignment of proteins contained in the PDB database with respect to the center of a bilayer. For this purpose, PDB_TM offers a transformation matrix which has to be applied to the structure of the original PDB model. OPM provides a different approach; it attaches dummy atom layers to the PDB model, indicating both sides of the membrane′s bilayer. Both approaches use special algorithms to compute the protein′s membrane alignment. Moreover, OPM offers a service which tries to attach the dummy layers to custom PDB files uploaded to the OPM server. Of course, these methods can be combined with the aforementioned Insertion methods. Both pre-alignment approaches can also be semi-automatically used in conjunction with the CELLmicrocosmos MembraneEditor discussed in the Membrane Modeling Tools section [15, 32].

### Self-assembly Methods

Another method is the self-assembly of membrane layers. It is theoretically a simple experiment for users of molecular simulation programs. A number of lipids are randomly placed in a virtual box using simple manual methods such as a text editor or molecular modeling program. These lipids are then immersed in water and then a molecular simulation is started. After a relatively short period (usually less than a nanosecond), the self-assembling process starts. The lipids often will try to form a vesicle. The head groups are hydrophilic and will orient themselves towards the water interface. As the tails are hydrophobic, they are directed towards the inner center of the vesicle.

Of course, this is only a very rough description and the success of this experiment depends on several factors, the applied force field, the duration, the temperature and pressure settings, etc. But there are three major problems. (1) The user is not able to exactly define the shape of the resulting structure. So it will be quite complicated to generate a bilayer model. (2) The challenging requirement on computational resources. If a well-established MD simulation package such as GROMACS is used, it is usually a good idea to use a computer or GPU cluster [7]. (3) Preparing such an *in silico* experiment is very time consuming if the user is not familiar with molecular simulation approaches.

Of course, there is also a big advantage: the resulting structure is in a perfect state for further simulations.

### Geometry-based Methods

To prevent the lipid′s distribution resulting in an overly artificial structure, it might be useful to begin by applying geometry-based methods during the generation process. This means that atom-based or shape-based collision detection is taken into account. The Geometry-based method is presented here as a serious alternative to the simple but fast grid-based method and the more realistic but complex methods described previously.

In our previous publication, we defined the assembly of a membrane layer as a *Two-and-a-half-dimensional Knapsack Problem (2.5D-KP)* [1]. The basics have to be explained to describe this term. For this purpose, the Lipid Packing Problem has to be examined from a computational perspective. One well-known computer science-related packing problem is the *Knapsack Problem (KP)*, which is formally defined as follows [33]:

1 $$\text{maximize}\;\sum_{i=1}^{n}v_{i}x_{i},\;\text{subject to constrain}\sum_{i=1}^{n}w_{i}x_{i}\leq W,x_{i}\in \left\{0,1,\ldots ,c_{i}\right\}$$

There are *n* kinds of items *x* with values of *v*_*i*_ and a weight of *w*_*i*_. And the overall weight of the given container is restricted to *W*. Here, the term *container* defines the distribution area. The number of copies *x*_*i*_ is restricted by the maximum value *c*_*i*_ which is a characteristic criterion for the *Bounded KP (BKP)*. Therefore, the knapsack′s area (for a 2D-KP) or volume (for a 3D-KP) is limited, in contrast with the *Unbounded KP (UKP)*, where *c*_*i*_ is defined as infinity. The values *v* and *w* are positive integer values *i* in the context of the LPP.

The original fluid mosaic membrane model already proposed the membrane as a two-dimensional liquid arrangement [2]. Now the question is, if this definition is sufficient in the context of LPP?

The knapsack′s weight *w*_*i*_ is often used to describe dimensionality. An extension of the KP is the so-called *Multidimensional KP* where knapsacks may feature multiple dimensions [33]. In the context of describing rectangular bilayers, the *two-dimensional* [9, 34] or *geometric* [35] *KP* class is appropriate *(2D-KP)*.

The basic KP is restricted to one container. But there is also another extension of the KP called the *Multiple KP (MKP)* which includes multiple related containers [36]. In the case of the LPP, a monolayer can be defined as one container and a bilayer as two containers. In contrast to the MKP, these two containers of a bilayer are not related. For example, in the case of the MembraneEditor, the modeling process strictly focuses on the geometric properties of the molecules, refusing chemophysical interactions as well as periodic boundary conditions. Therefore, both layers are regarded as being strictly divided by the hydrophobic core in the center of the bilayer. Of course it is known that *in vivo* interactions between both layers steadily occur, for example in case of cholesterol flip-flops [24].

Now, the different variables of Equation 1 have to be assigned to the LPP. The container represents one membrane layer whereas the different items are the molecules. For the regular LPP, many items of relatively few differing figures or shapes exist [36]. The items are the lipid models and their frequency is defined by the lipid ratio. In the case of the LPP, the weight w_i_ is represented by the width (along the x-axis) and length (along the z-axis) of the molecule. This might be confusing for the experimentalist, because a biological lipid has molecular weight. But this weight is not relevant for a strictly geometry-based approach. But of course, the height and width are important, because a larger lipid needs more space in the knapsack. The value v_i_ is one, because each molecule is counted as one and added to the overall number of lipids. If different lipid types are part of the layer (for example, 80% phospholipid and 20% cholesterol), c_i_ is restricted by the lipid percentages and BKP would be the appropriate class. In this case, the lipid will not be placed in a free space if the actual percent value for the according lipid type is equal or larger than the lipid percentages. By contrast, UKP is the appropriate definition if the according layer contains only one lipid type. Then, c_i_ is infinity.

Many classical KP solutions are not applicable to LPP, because the item′s orientation is also fixed or it is only altered in 90° steps [9, 34]. This is not possible if the items are represented by lipids. Packing problems such as KP are NP-hard [37, 38]. Various alternative non-exhaustive solutions exist, for example: approximate algorithms, greedy algorithms, and heuristic algorithms [33]. A single good solution is sufficient for many application cases in contrast to an exhaustive one which is usually not computable during an appropriate period of time.

Finally, the term 2D-KP should be reevaluated. For strictly two-dimensional problems – for example, the distribution of boxes or spheres on a two-dimensional area – this definition is correct. Even if the shape of the lipid on a two-dimensional plane is irregular – which is almost always the case – it is still a two-dimensional packing problem. But in the case of distributing three-dimensional objects like molecules, the collision detection should also operate in three dimensions, whereas the movement of the lipid is restricted to the two-dimensional plane.

Therefore, the term 2.5D-KP was introduced in our previous work [1]. It was shown, that the 2.5D-KP is the appropriate problem definition for the geometrical packing of lipids on a plane.

## Discussion

The last two sections showed that the borders between the different methods are floating. They are often combined. For example: (1) The lipid′s layer is created using a Grid-based method and then a replacement or insertion method is applied. (2) Lipids are initially substituted by simple structures but the proteins remain as atomic structures, or, inversely (3) the proteins are temporarily represented by shapes and the lipids are handled as atomic structures.

The traditional generation processes are naturally quite complex. They are often not straight-forward. The previous examples showed that often multiple minimization attempts are needed to generate a membrane model usable for the following simulations steps or during the minimization of the molecules′ radii (which have to increase to perform the alignment and so forth).

Another problematic aspect of most traditional Membrane Modeling Methods is that the generation protocols found in many articles are vague and therefore the results are not reproducible. Of course, it is an ongoing debate in science if all methods described in a publication must be reproducible. The following example is one among a thousand which illustrate this aspect. (In no way should the quality of the following publication be doubted as this sentence is only shown for illustrative purposes.)

*“The configurations were assembled as a set of rigid units, with each GA or DMPC (with their primary waters) being translated and rotated in a systematic search for an optimum packing”* [39]

But how do the traditional Membrane Modeling Methods apply to the KP? The initial problem is the same; an area has to be filled with lipids. Moreover, the replacement method utilizing spheres as lipid-placeholders can be interpreted as a typical 2D-UKP. The knapsack is unbounded if the spheres are all of the same type. It is a two-dimensional packing problem, because the movement of the spheres is restricted to the X/Z-plane and the packing can be reduced to the two-dimensional area where the radii of the spheres are at a maximum size. After replacing the spheres with the lipid′s atomic structure, different methods are used to remove collisions. Methods like the minimization and equilibration, which are normally used for the replacement methods, are not typical packing problems, since chemophysical methods are applied here which are not restricted to geometrical issues. Still, the Replacement methods could be seen as a packing problem but with different new criteria. But these criteria are not restricted to the shape. They apply to every single atom during the minimization processes. Every atom can move in different directions. But regular packing problems, generally and more particularly the KP, take stable shapes or outer boundaries into account. The LPP is defined for stiff lipid structures and the KP optimization takes advantage of the fact that the lipid′s structure does not change during the distribution process. The criterion “many items of relatively few different figures or shapes” would not be met in this case [36].

## Membrane Modeling Tools

Five tools will be briefly introduced providing capabilities to generate membranes without the need for computing complex molecular interactions on a local computer. In addition, these tools do not assume chemophysical expert knowledge and the regular computer user is able to adapt the tool′s workflow in an appropriate time frame. Each of the tools discussed here must be able to export structures in a format which is compatible with MD packages. And the regular export format which is supported by all introduced tools is the *Protein Data Bank (PDB) format* [40]. Although the name of this format suggests that is only appropriate for proteins, nowadays it is the standard format for all applications working with three-dimensional molecular structures. It is, for example, possible to obtain lipid structures based on molecular assemblies from the PDB repository using the websites HIC-UP or Ligand Expo [41–43]:http://xray.bmc.uu.se/hicup/ http://ligand-expo.rcsb.org

It has to be emphasized that these are tools solving the Membrane Packing Problem. In the section discussing the Membrane Modeling Methods, there were also tools listed which explicitly solve the Protein Packing Problem (ProtSqueeze, g_membed,, and GRIFFIN, for example).

### ChemSW^®^ ChemSite Pro^®^

Chemsite Pro^®^ (Version 7) from the company ChemSW^®^ is the second commercial tool besides MOE. This package is also to model and simulate molecular structures in addition to other functionalities [44]. It provides an intuitive approach for modeling membranes. This program also comes packaged with the MolSuite™ which provides quantum mechanical simulations and a database containing different molecular structures.

The module *Build Lipid Membrane* of ChemSite Pro is interesting in the context of this review. It provides a number of simple options to create a lipid membrane. First, the user can decide if a symmetric or asymmetric monolayer or bilayer should be created. Then, the width of the quadratic-shaped membrane is defined by the number of lipids along each edge multiplied by the user-defined distance between each lipid. It is not possible to generate membranes with different side lengths. Obviously, this is a Grid-based approach.

It is possible to apply a simple solvation to the membrane by defining the number of water layers at each side of the bilayer as the addition of water layers is often needed for MD simulations. ChemSite Pro places parallel rows of water layers beneath the heads of the highest lipids. Therefore, distances will appear between small lipids like cholesterol and the water layer. The resulting membrane models will be problematic for a sophisticated simulation, because the layer might collapse when the water molecules penetrate the bilayer and they are found everywhere in the system, even between the two lipid layers in the hydrophobic area.

Now, the lipids have to be defined. Theoretically an unlimited number of lipid types can be added, each represented by a name and a percent value. In case the membrane is not symmetrical and a bilayer was chosen, the values for each lipid type have to be defined for both sides and it is possible to generate asymmetric bilayers. Finally, the “Build” button has to be pressed and the membrane is created. If the contained lipids are charged, counter ions are automatically added to maintain a state of equilibrium.

The disadvantages of the grid-like structure have been previously discussed. Another problem is that the resulting membrane is not a mixed bilayer because the different lipid types are placed iteratively; the starting rows are filled with lipid type1 until the percentages are reached, and then the following rows are filled with lipid type 2 and so forth.

ChemSite uses a native library format for lipids, called *lib*. It contains the coordinate information and some further information regarding charges. It is not directly possible to use PDB files to generate these membranes. It is, however, a simple task to translate PDB to lib files by using ChemSite Pro, to re-import them and to integrate them into the membrane. The lipid library which comes with ChemSite Pro contains six different types, four phospholipids, one phosphatic acid and one cholesterol.

Moreover, ChemSite Pro includes a nice tool to create a large number of custom lipids, the *Lipid Builder*.

But there are also some disadvantages. The program exhibits low performance. For example, if a membrane with 100 lipids is explored, juddering occurs unless a powerful computer is used. Moreover, the layout of the GUI is outdated and there seems to be no community supporting or using this tool. Because of the very simple generation method, the resulting membranes exhibit an extremely high energy state. Therefore, a very long minimization run is needed before the MD production run can be started. This is problematic as it it not possible to use a cluster system for the minimization process with ChemSite Pro and it must be computed on a desktop system.

### VMD Membrane-Plugin

*VMD (Visual Molecular Dynamics)* is a popular program to visualize, analyze, and edit molecular structures and simulation files [45]. It is also possible to simulate membranes by connecting external packages like NAMD [6]. VMD also supports plugins, such as the “Membrane” plug-in which was developed by Ilya Balabin, on-line since 2006 [46]. It is the simplest tool discussed here, but the fact that it is still part of the actual VMD release 1.9.1 shows that it is accessible to a large community. The simple syntax of the membrane plugin′s command line tool is as follows:

membrane -l <lipid_name> -x <size_in_X> -y <size_in_Y> {-o <output_prefix > }

The lipid type is defined behind the *-l*, where there are only two options: POPC (a phosphatidylcholine) and POPE (phosphatidylethanolamine). Only bilayers containing one single lipid type are possible. The lipids feature outstretched tails to improve the packing quality. The size of the rectangular membrane is defined by the variables *-x* and *-y*. The variable *-o* defines the prefix for the two membrane output files. These are a PDB file (containing the coordinate information) and a *PSF file* (containing force-field-specific structural information of the membrane model).

The membrane is generated by combining hexagonal patches of shortly equilibrated solvated POPC or POPE bilayers. The solvation is based on the *Solvate plugin* from Grubmüller and Groll which generates an irregularly-shaped disordered water layer [47]. This resulting structure can be directly used via VMD for MD simulations.

The tool, packaged with VMD, is found at: http://www.ks.uiuc.edu/Research/vmd/

### CHARMM-GUI Membrane Builder

*CHARMM (Chemistry at HARvard Macromolecular Mechanics)* is a highly established program package providing different tools and force fields for the minimization and simulation of different molecular structures [8, 23]. Unlike the free MD tools such as GROMACS, the user has to obtain academic or commercial licenses.

The *CHARMM-GUI* initiative is quite new and it may be used free of charge. Here, the version 1.2 is discussed. As denoted by the name, the target group is mainly users of the CHARMM package. CHARMM-GUI provides a set of tools which should simplify the process of generating starting structures for MD simulations [48]. The usage is quite simple because CHARMM-GUI can be directly accessed via the website [49]: http://www.charmm-gui.org

For this mini review, only the *Membrane Builder* will be discussed. It is part of a collection of tools found in the website section called “Input Generator”. At the moment, it is only possible to generate bilayers; monolayers are not possible. For homogeneous bilayers, six lipid types are available (DMPC, DPPC, DOPC, POPC, DLPE and POPE) and for heterogeneous bilayers, 32 lipid types can be used (incl. cholesterol and different sphingolipids). Each lipid type in this library is represented by 2,000 different conformations extracted from MD simulated bilayers [50]. It is possible to use a quadratic as well as hexagonal shape.

Now, the generation process for “Membrane Only Systems” will be discussed. It is subdivided into five different steps.

Step 0) In the preliminary step, the conformation of the bilayer is defined, including the shape, the thickness of the water layer, the initial estimate for the rectangular size and whether or not ratios or numbers should be used as a reference point. All available lipids – depending on the type of membrane – are listed and the user can choose the absolute or the percentaged distribution values for each bilayer side. It is also possible to edit the initial guess for the surface area for each lipid type. There is a button which can be used to evaluate the predicted properties of the membrane model which will be generated as follows:

Step 1) The initial files are generated and the properties of the files are now shown. For the assembling process the user can choose between the Insertion method (only available in homogeneous bilayers, see the corresponding Membrane Modeling Method of Jo, Kim and Im) or the Replacement method, from Woolf and Roux. This latter method places pseudo atoms onto the membrane area [19]. After the initial distribution has finished, the pseudo atoms are replaced by molecular structures of the aforementioned lipid library.

The insertion method depends on a library of pre-equilibrated bilayers which are restricted to a maximum size of 90x90 Å^2^, containing maximal 256 lipids. For the Membrane Builder, the rapid Insertion method should be used for proteins of regular and cylindrical shape and for all other cases the Replacement method. If charged molecules were found, the program is able to inhibit counter ions and the user may choose between different ion types [31].

Step 2) shows the previously-created membrane components; the ions, a newly generated water box and the lipid-holding layer. It is now possible to download and/or to look at a preview of the PDB structure of the lipid layer using an integrated Java applet called MarvinSpace (Version 5.4) from ChemAxon Ltd. Note; this tool seems to have problems with larger membrane files.

Step 3) provides information and editable options regarding the equilibration process, the temperature, the ensemble type and the surface tension.

Step 4) enables the user to download the resulting files which can be used for further equilibration and simulation with CHARMM. These files are also compatible with NAMD simulations which can be used with VMD (discussed in the previous section).

The workflow and the results provided by CHARMM-GUI are quite powerful, particularly for users of the commercial CHARMM software package. It also provides output files compatible with NAMD, so users of this software package might be interested in using this tool. Although initially planned, CHARMM-GUI does not currently provide simulation files compatible with GROMACS [23].

An important and unique feature of the Membrane Builder in comparison to the other tools discussed here is the computation of lipid′s surface areas by using Voronoi tessellation based on Pandit et al. [51]. These estimations are appropriate criteria for judging the space occupied by a lipid model. In the case of heterogeneous membrane layers, this method is more accurate than just computing the average area per lipid, because the shape among different lipid types strongly differs.

Although the generation process is well-described in publications, the source code is not available. Users of Open Source software, such as GROMACS, prefer to use software providing access to all data of the simulation process. Therefore, it might be undesireable to use a module in the modeling and simulation process which shows similarities to a black box.

In terms of reproducibility there are also some drawbacks. First, the program is only available via Internet and reproduction of the results is only possible as long as the website is accessible and the provided methods are not changed by the developers. While the use of the Membrane Builder is presently free of charge, further simulation using the CHARMM simulation package requires a paid license.

Because it is not possible to import custom lipid models, many MD packages like GROMACS will probably require the additional manipulation of the PDB membrane created by the Membrane Builder by using the Replacement method for example.

The generation methods do not take collision-detection of covalent or van der Waals radii into account. Therefore, collisions occurring after applying the Replacement method have to be eliminated by more expensive minimization and other special methods described in the original publication of the Membrane Builder [50].

### Packmol

In the collection of tools now presented, *Packmol* is the only tool which is exclusively available as a command line tool. Therefore, its handling will not be easily accessible to a number of users. But it has to be discussed because it is quite powerful as it is able to generate molecular structures of different shapes such as spheres, cylinders, planes and/or boxes by applying geometry-based methods. As with most other tools discussed here, it supports homogeneous as well as heterogeneous membrane layers. The old version of Packmol has already been used for many publications since 2003 [52]. Published in 2009, the new version supports a more effective distribution algorithm combined with the ability to parallelize the computation [53].

It uses geometry-based methods implemented using a special packing algorithm called GENCAN [54]. It is able to solve the previously introduced 2.5D-KP, but it is also able to generate membrane structures with complex three-dimensional shapes like vesicles. This tool is particularly powerful if small molecules need to be packed into three-dimensional volumes defined of various shape.

However, Packmol is also often used for generating rectangular homogeneous membranes. Moreover, there are also a small number of publications found which generate heterogeneous membranes with Packmol, e.g., Hall et al. [55]. For this purpose, the user exactly calculates the absolute number of lipids which will be placed inside the membrane layer. The lipid models are manipulated to be compatible with Packmol. Since the position and orientation along the vertical axis of the different lipid models should match they are pre-aligned.

Packmol is invoked by a customized script. This script contains all information about how the membrane has to be constructed. For new users it might be a good idea to search for an existing script and customize it. The positioning of lipids can be defined via constraints. It is even possible to restrict the vertical position of the headgroups to be placed beneath a predefined plane. This feature is quite powerful but requires the user to have an exact idea of the coordinate space. Therefore, mathematical abstract thinking is a prerequisite for the correct use of Packmol.

The packing process integrated in Packmol may result in a number of problems during the generation of a membrane. If the structures are too complex, the packing optimum might never be reached. To find out which layer sizes and lipid ratios are compatible, the user often has to find out by trial and error. In addition, the resulting structure has to be equilibrated if MD packages like GROMACS, CHARMM or NAMD are used. Packmol does not offer any visualization capabilities. But of course, for this purpose external tools like Jmol can be used [56]. The program is located at:http://www.ime.unicamp.br/∼martinez/packmol/

### CELLmicrocosmos 2.2 MembraneEditor (CmME)

The *CELLmicrocosmos 2.2 MembraneEdior (CmME)* is the newest of the tools represented here. It was developed for the fast and user-friendly modeling of membranes. It is the only tool strictly applying geometry-based methods to the LPP. In contrast to CHARMM-GUI Membrane Builder, it does not support the equilibration of the membrane. The philosophy of the CmME is that the equilibration should be done directly with the tool of choice, like GROMACS or NAMD. In our publication from 2011 it was shown that these methods produce good results by using GROMACS [1]. Moreover, we are currently developing the *GMX-Plugin* which should be a bridge between CmME and GROMACS.

A number of Lipid Packing Algorithms (LPA) provided by CmME solves the 2.5D-KP. Rectangular, homogeneous or heterogeneous monolayers as well as bilayers can be computed using different plug-in algorithms. It is possible to use percentages to define the composition of the membrane as well as absolute values. Micro domains/lipid rafts can be drawn by hand or shaped as rectangles or ellipses. It is also possible to generate stacked membranes using CmME.

Every valid PDB file can be imported and defined as a lipids or protein. Lipids are automatically aligned perpendicular to the lipid layer. The semi-automatic placement of proteins which solve the PPP by using PDB_TM and OPM was already mentioned in the Membrane Modeling Methods section as well as in the original publication of the MembraneEditor [1]. There are two ways of combining a protein and a membrane. First, it is possible to use the Insertion method. In this case, the lipids are distributed on the plane, the protein is inserted and then the intersecting lipids are removed. Problems for transmembrane proteins arising from this procedure were already discussed in the Insertion method section. In the other case, first the protein/s is/are placed onto the empty membrane area and then the lipids are distributed around the protein using the Lipid Packing Algorithms. For transmembrane proteins, this is the procedure of choice. For extrinsic proteins, the Insertion procedure should be used.

CmME features WYSIWYG (What You See Is What You Get). The lipids are visually represented by shapes and their distribution is also directly visualized. Therefore, associations with the replacement method are possible. But this does not completely apply to the approaches of CmME, because the collision detection may take the visual shapes and/or the atomic structures into account. Moreover, the structure of the atomic structure can also be directly visualized with CmME. But it is advised to use Jmol – which is bundled with CmME – for this purpose.

To judge the packing density, CmME computes the average overall surface area. In contrast to CHARMM-GUI Membrane Builder, this method is not based on Voronoi tessellation and is therefore less accurate. But it provides initial insight into the quality of the LPAs. Moreover, we are currently developing a program which provides extensive analysis functionalities for lipid membranes by using Voronoi tessellation.

It was previously mentioned that Packmol supports the generation of vesicles. For CmME, there is also an initial version of a Vesicle Builder available at the same website. Here CmME can be directly downloaded and started as Java Web Start application:http://Cm2.CELLmicrocosmos.org/

## Conclusions

Five different Membrane Modeling Method categories were introduced: the Grid-based methods, the Replacement methods, the Insertion methods, the Self-assembly methods and the Geometry-based methods. By examining these methods it was shown that the distinction of Membrane Packing Problems into 1) Protein packing Problems and 2) Lipid Packing Problems is reasonable, because in many cases only one of these problems was addressed. Furthermore, if proteins and lipids are combined in a system, the packing processes of both molecule types usually differ significantly.

Then, a number of Membrane Modeling Tools were discussed which are all able to handle the Membrane Packing Problem and which implement at least one of the aforementioned Membrane Modeling Methods.

*MOE* ^*®*^ was used to present a Grid-based method which can also be applied by using the scripting capability of other tools. This approach is interesting for all those users who, 1) already know their program of choice quite well and, 2) are familiar with the scripting language. For all other users it will be more efficient to use one of the following approaches.

*ChemSite Pro* ^*®*^ from ChemSW^®^ might be good choice for those people using the MolSuite^™^ developed by the same company. However, this grid-based approach is quite simple and there are gaps between the placed lipids. Moreover, different lipid types in heterogeneous membranes are not mixed. Therefore, model membranes which should be simulated – especially those with external packages – should be modeled using one of the following tools.

**Table 1:** The complete comparison of all membrane modeling tools discussed in this work (Charmm GUI MB: Charmm GUI Membrane Builder; VMD MP: VMD Membrane Plugin; GMX: The Gromacs-Plugin for the MembraneEditor; VB: The Vesicle Builder Plugin for the MembraneEditor).

Category	Feature	Charmm GUI MB	Chem Site Pro	CmME^1^	MOE+Script	Packmol	VMD MP
Availability	Standalone		X	X	X		X
	Command line tool					X	
	Web service						
	Website	X					
	Web Start			X			
	Source Code			X		X	X
	Licenses(educational license fee)		commer-cial	GPL3	commer-cial	GPL3	UIUC Open Source

Computational Acceleration	Multi-threading Parallelization					X	

Direct PDB Database Connection		X		X	X		

Formats	GRO						X
	native format		X	X	X		
	PDB	X	X	X	X	X	X

Membrane Modeling Methods	Grid-based		X		X		X
	Replacement	X		X			
	Insertion	X		X			
	Self-assembly						
	Geometry-based			X		X	

Membrane Shapes	Free			X		X	
	Hexagonal Shape	X					
	Quadratic (X = Z)	X	X	X	X	X	
	Rectangular (X#x2260;Z)			X	X	X	X
	Vesicles			(VB)		X	

Libraries	Lipid library (>2 lipid types)	X	X	X			
	Lipid library compatible to MD	X	X	(GMX)			

Lipid Area Computation	Average Surface Area per Lipid			X			
	Surface Area by Voronoi Tessellation	X					

Features	Asymmetry	X	X	X		X	
	Atom-based Molecule Editor		X	X	X		X
	Automatic Alignment of imported Lipids			X			
	Bilayers	X	X	X	X	X	X
	Collision-Detection			X		X	
	Counter ions support	X		(GMX)			
	Lipid Packing Density	X		X			
	Heterogeneity	X	(X)	X		X	
	Lipid Ratio: Absolute	X		X	X	X	X
	Lipid Ratio: Relative	X	X	X			
	Mono-layers		X	X	X	X	
	Multi-layers			X		X	
	Protocol			X			
	Raft Support			X		X	
	Reproducibility			X			
	Water Layer Build	X	X	(GMX)	X	X	
	WYSIWYG		X	X	X		X

Operating System	Linux	X		X	X	X	X
	Mac OS X	X		X	X	X	X
	Windows XP, VISTA, 7	X	X	X	X	X	X

Pipelines	Ext. Simulation Package	X	X	(GMX)	X		X
	Ext. Visualization Package	X	X	(Jmol)			X

Programming	Scripting			X	X	X	X
	Scripting at Runtime			X			X

Simulation	Equilibration	(X)		(GMX)	X		X
	Minimization	(X)	X	(GMX)	X	X	X
	Simulation		X	(GMX)	X		X

Visualization	Graph Visualizations			X	X		
	Live Distribution Visualization			X			
	Atomic Structure View	X	X	X	X		X
	Secondary Structure View			(Jmol)	X		X
	Ray-tracing		x				
	Runtime Graphs			X			
	Stereo Support			X	X		X

1 GMX = GMX-Plugin (in development); Jmol = Jmol library (directly included in CmME); VB = VesicleBuilder-Plugin (in development

### Membrane Modeling Tools Overview

The *VMD Membrane-Plugin* is the right choice for modeling a homogeneous POPC or POPE bilayer membrane. The biggest advantage is that it is part of the popular VMD and nearly every member of the modeling community will have this tool on the computer. This plugin is the first choice if the membrane should be simulated with NAMD and there is no need for other lipid types.

*CHARMM-GUI Membrane Builder* is a very powerful website-based tool. It applies the Replacement as well as the Insertion method (only for homogeneous bilayers) to solve the Lipid Packing Problem. It usually will be the first choice for all users of the CHARMM simulation package. It provides a large number of regularly-used lipid types. However, it is not possible to import custom lipid types. Another small drawback is that the shapes of the membranes have to be quadratic. For users of other simulation packages – especially Open Source tools – one should think twice about the use of the Membrane Builder because the source code is not available and the tool is optimized for use with CHARMM. But the additional option to generate NAMD-compatible files is a step in the right direction. The Membrane Builder is also well-described in its original publication [50].

Finally, the two Lipid Packing approaches contributed by the Open Source community have to be summarized.

*Packmol* is a very powerful and versatile package which is able to generate molecular structures of many different shapes using geometry-based methods. One application case is the generation of lipid layers. In contrast with the following, it is a command-line tool providing no visualization and is invoked with a script. The writing of this script needs the user to have a clear picture of the geometrical properties of the molecules used and the resulting structure. The lipids have to be pre-aligned and the composition of the membrane has to be defined by constraints. Packmol directly tries to minimize the resulting structure by using its custom methods. In any case, it will be the first choice if the user needs to generate structures consisting of very small molecules such as water, which should be three-dimensionally distributed [53].

*CELLmicrocosmos 2.2 MembraneEditor (CmME)* was developed from the start to generate heterogeneous membrane patches in a fast and easy way by using geometry-based methods. For a fast modeling and evaluation of a membrane composition it should be the first choice. Of course, if chemophysical properties of a membrane should be evaluated, the resulting structures should be minimized, equilibrated and simulated by external, well-established simulation environments like GROMACS. We recently showed that structures generated with CmME are compatible with MD simulations of GROMACS. A first version of a Vesicle Builder is also available. The Java Web Start version of this tool enables quick installation of this tool [1].

As the title of this mini review indicates, the detailed explanation and evaluation of the Membrane Packing Problem lies beyond the scope of this exposition and the complex methodologies are only roughly described. Moreover, this short review does not claim to list all known Membrane Packing approaches.

## References

[1] Sommer B, Dingersen T, Gamroth C, Schneider SE, Rubert S, et al. (2011). CELLmicrocosmos 2.2 MembraneEditor: a modular interactive shape-based software approach to solve heterogeneous Membrane Packing Problems. Journal of Chemical Information and Modeling 5: 1165–1182 21504163 10.1021/ci1003619

[2] Singer SJ, Nicolson GL(1972) The fluid mosaic model of the structure of cell membranes Science (Washington, DC, US) 175: 720–731 10.1126/science.175.4023.720 4333397

[3] Bennett WFD, MacCallum JL, Hinner MJ, Marrink SJ, Tieleman DP (2009). Molecular view of cholesterol flip-flop and chemical potential in different membrane environments. J Am Chem Soc 131: 12714–12720 19673519 10.1021/ja903529f

[4] Lingwood D, Simons K(2010) Lipid rafts as a membrane-organizing principle Science (Washington, DC, US) 327: 46–50 10.1126/science.1174621 20044567

[5] Park K, Götz AW, Walker RC, Paesani F (2012). Application of adaptive QM/MM methods to molecular dynamics simulations of aqueous systems. J Chem Theory Comput 8: 2868–2877 10.1021/ct300331f 26592126

[6] Phillips JC, Braun R, Wang W, Gumbart J, Tajkhorshid E et al. (2005). Scalable molecular dynamics with NAMD. J Comput Chem 26: 1781–1802 16222654 10.1002/jcc.20289 PMC2486339

[7] Hess B, Kutzner C, Van der Spoel D, Lindahl E (2008). Gromacs 4: Algorithms for highly efficient, load-balanced, and scalable molecular simulation. J Chem Theory Comput 4: 435–447 10.1021/ct700301q 26620784

[8] Brooks BR, Brooks CL III, Mackerell AD Jr , Nilsson L, Petrella RJ, et al. (2009). CHARMM: the biomolecular simulation program. J Comput Chem 30: 1545–1614 19444816 10.1002/jcc.21287 PMC2810661

[9] Lodi A, Martello S, Monaci M (2002). Two-dimensional packing problems: A survey. Eur J Oper Res 141: 241–252

[10] Wottawa M(1996) Struktur und algorithmische Behandlung von paraxisorientierten dreidimensionalen Packungsproblemen [Doctorate Thesis]. Köln, Germany: University of Cologne

[11] Tusnády GE, Dosztányi Z, Simon I (2005). TMDET: web server for detecting transmembrane regions of proteins by using their 3D coordinates. Bioinformatics 21: 1276 15539454 10.1093/bioinformatics/bti121

[12] Kandt C, Ash WL, Peter Tieleman D (2007). Setting up and running molecular dynamics simulations of membrane proteins. Methods 41: 475–488 17367719 10.1016/j.ymeth.2006.08.006

[13] Wolf MG, Hoefling M, Aponte-Santamaría C, Grubmüller H, Groenhof G (2010). g_membed: Efficient insertion of a membrane protein into an equilibrated lipid bilayer with minimal perturbation. J Comput Chem 31: 2169–2174 20336801 10.1002/jcc.21507

[14] Staritzbichler R, Anselmi C, Forrest LR, Faraldo-Gómez JD (2011). GRIFFIN: A Versatile Methodology for Optimization of Protein- Lipid Interfaces for Membrane Protein Simulations. J Chem Theory Comput 7: 1167–1176 10.1021/ct100576m PMC3972769 24707227

[15] Lomize MA, Pogozheva ID, Joo H, Mosberg HI, Lomize AL (2012). OPM database and PPM web server: resources for positioning of proteins in membranes. Nucleic Acids Res 40: D370–D376 21890895 10.1093/nar/gkr703 PMC3245162

[16] Krüger J, Fischer WB (2008). Exploring the conformational space of Vpu from HIV-1: a versatile adaptable protein. J Comput Chem 29: 2416–2424 18432615 10.1002/jcc.20986

[17] Molecular Operating Environment (MOE) (2010). Montreal, Canada: Chemical Computing Group Inc

[18] Pastor RW, Venable RM, Karplus M (1991). Model for the structure of the lipid bilayer. Proc Natl Acad Sci USA 88: 892–896 1992480 10.1073/pnas.88.3.892 PMC50920

[19] Woolf TB, Roux B (1996). Structure, energetics, and dynamics of lipid–protein interactions: a molecular dynamics study of the gramicidin A channel in a DMPC bilayer. Proteins 24: 92–114 8628736 10.1002/(SICI)1097-0134(199601)24:1<92::AID-PROT7>3.0.CO;2-Q

[20] Petrache HI, Grossfield A, MacKenzie KR, Engelman DM, Woolf TB (2000). Modulation of glycophorin A transmembrane helix interactions by lipid bilayers: molecular dynamics calculations1. J Mol Biol 302: 727–746 10986130 10.1006/jmbi.2000.4072

[21] Zidar J, Merzel F, Hodošcěk M, Rebolj K, Sepčić K, et al. (2009). Liquid-ordered phase formation in cholesterol/sphingomyelin bilayers: all-atom molecular dynamics simulations. J Phys Chem B 113: 15795–15802 19929009 10.1021/jp907138h

[22] Brooks BR, Bruccoleri RE, Olafson BD (1983). CHARMM: a program for macromolecular energy, minimization, and dynamics calculations. J Comput Chem 4: 187–217

[23] Venable RM, Zhang Y, Hardy BJ, Pastor RW(1993) Molecular dynamics simulations of a lipid bilayer and of hexadecane: an investigation of membrane fluidity Science (Washington, DC, US) 262: 223–226 10.1126/science.8211140 8211140

[24] Woolf TB, Roux B (1994). Molecular dynamics simulation of the gramicidin channel in a phospholipid bilayer. Proc Natl Acad Sci USA 91: 11631–11635 7526400 10.1073/pnas.91.24.11631 PMC45285

[25] Shen L, Bassolino D, Stouch T (1997). Transmembrane helix structure, dynamics, and interactions: multi-nanosecond molecular dynamics simulations. Biophys J 73: 3–20 9199766 10.1016/S0006-3495(97)78042-1 PMC1180903

[26] Tieleman DP, Berendsen HJC (1998). A molecular dynamics study of the pores formed by Escherichia coli OmpF porin in a fully hydrated palmitoyloleoylphosphatidylcholine bilayer. Biophys J 74: 2786–2801 9635733 10.1016/S0006-3495(98)77986-X PMC1299620

[27] Faraldo-Gomez JD, Smith GR, Sansom MS (2002). Setting up and optimization of membrane protein simulations. Eur Biophys J 31: 217–227 12029334 10.1007/s00249-002-0207-5

[28] Brasseur R(1990). Molecular Description of Biological Membrane Components by Computer Aided Conformational Analysis. CRC Vol. 1 pp. 203–219

[29] Deleu M, Bouffioux O, Razafindralambo H, Paquot M, Hbid C, et al. (2003). Interaction of surfactin with membranes: a computational approach. Langmuir 19: 3377–3385

[30] Yesylevskyy SO (2007). ProtSqueeze: simple and effective automated tool for setting up membrane protein simulations. J Chem Inf Model 47: 1986–1994 17649971 10.1021/ci600553y

[31] Jo S, Kim T, Im W (2007). Automated builder and database of protein/membrane complexes for molecular dynamics simulations. PloS one 2: e880 17849009 10.1371/journal.pone.0000880 PMC1963319

[32] Tusnády GE, Dosztányi Z, Simon I (2005). PDB_TM: selection and membrane localization of transmembrane proteins in the protein data bank. Nucleic Acids Res 33: D275–D278 15608195 10.1093/nar/gki002 PMC539956

[33] Kellerer H, Pferschy U, Pisinger D(2004) Knapsack problems Berlin, Germany: Springer Verlag

[34] Dyckhoff H, Finke U (1992) Cutting and packing in production and distribution: a typology and bibliography Heidelberg, Germany: Physica-Verlag

[35] Cagan J (1994). Shape annealing solution to the constrained geometric knapsack problem. Comput Aided Design 26: 763–770

[36] Dyckhoff H (1990). A typology of cutting and packing problems. Eur J Oper Res 44: 145–159

[37] Martello S, Toth P(1990) Knapsack problems: algorithms and computer implementations John Wiley & Sons, Inc

[38] Kaystha S, Agarwal S(2010) Greedy genetic algorithm to Bounded Knapsack Problem. 3rd IEEE International Conference on Computer Science and Information Technology (ICCSIT) 2010 IEEE, Vol. 6 pp. 301–305

[39] Woolf TB, Roux B (1994). Conformational flexibility of o-phosphorylcholine and o-phosphorylethanolamine: a molecular dynamics study of solvation effects. J Am Chem Soc 116: 5916–5926

[40] Berman HM, Westbrook J, Feng Z, Gilliland G, Bhat TN (2000). The Protein Data Bank. Nucleic Acids Res 28: 235–242 10592235 10.1093/nar/28.1.235 PMC102472

[41] Kleywegt GJ, Jones TA (1998). Databases in protein crystallography. Acta Crystallogr. Sect D: Biol Crystallogr 54: 1119–1131 10.1107/s0907444998007100 10089488

[42] Feng Z, Chen L, Maddula H, Akcan O, Oughtred R, et al. (2004). Ligand Depot: a data warehouse for ligands bound to macromolecules. Bioinformatics 20: 2153 15059838 10.1093/bioinformatics/bth214

[43] HIC-Up (2011). Available: http://xray.bmc.uu.se/hicup/. Accessed August 30, 2011

[44] ChemSite Pro (2010). Fairfield, USA: ChemSW

[45] Humphrey W, Dalke A, Schulten K (1996). VMD: Visual Molecular Dynamics. J Mol Graph 14: 33–38 8744570 10.1016/0263-7855(96)00018-5

[46] Balabin I (2006) Membrane Plug-in, Version 1.1. http://www.ks.uiuc.edu/Research/vmd/plugins/membrane/ (accessed October 1 2010).

[47] Grubmüller H, Groll V(1996) Solvate - Max-Planck-Institut für biophysikalische Chemie, Göttingen. Available: http://www.mpibpc.mpg.de/home/grubmueller/downloads/solvate/index.html. Accessed August 2, 2011.

[48] Jo S, Kim T, Iyer VG, Im W (2008). CHARMM GUI: a web-based graphical user interface for CHARMM. Journal of computational chemistry 29: 1859–1865 18351591 10.1002/jcc.20945

[49] CHARMM-GUI (2011). Available: http://www.charmm-gui.org/. Accessed 4 August 2011.

[50] Jo S, Lim J, Klauda J, Im W (2009). CHARMM-GUI Membrane Builder for Mixed Bilayers and Its Application to Yeast Membranes. Biophys J 97: 50–58 19580743 10.1016/j.bpj.2009.04.013 PMC2711372

[51] Pandit SA, Vasudevan S, Chiu SW, Jay Mashl R, Jakobsson E, et al. (2004). Sphingomyelin-cholesterol domains in phospholipid membranes: atomistic simulation. Biophys J 87: 1092–1100 15298913 10.1529/biophysj.104.041939 PMC1304449

[52] Martínez JM, Martínez L (2003). Packing optimization for automated generation of complex system's initial configurations for molecular dynamics and docking. J Comput Chem 24: 819–825 12692791 10.1002/jcc.10216

[53] Martínez L, Andrade R, Birgin EG, Martínez JM (2009). Packmol: A package for building initial configurations for molecular dynamics simulations. J Comput Chem 30: 2157–2164 19229944 10.1002/jcc.21224

[54] Birgin EG, Mario Martínez J (2002). Large-scale active-set box-constrained optimization method with spectral projected gradients. Comput Optim Appl 23: 101–125

[55] Hall A, Róg T, Karttunen M, Vattulainen I (2010). Role of glycolipids in lipid rafts: a view through atomistic molecular dynamics simulations with galactosylceramide. J Phys Chem B 114: 7797–7807 20496924 10.1021/jp912175d

[56] Herrez A(2007) How to use Jmol to study and present molecular structures Morrisville, USA: Lulu Enterprises

